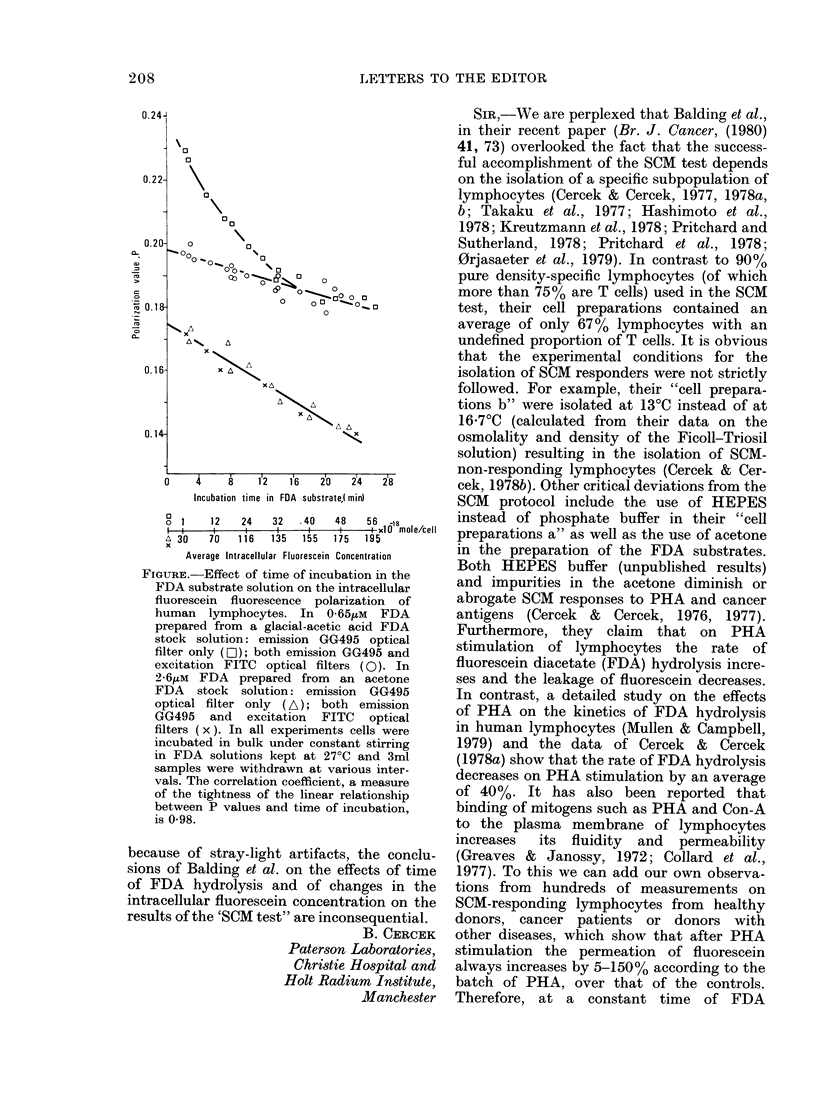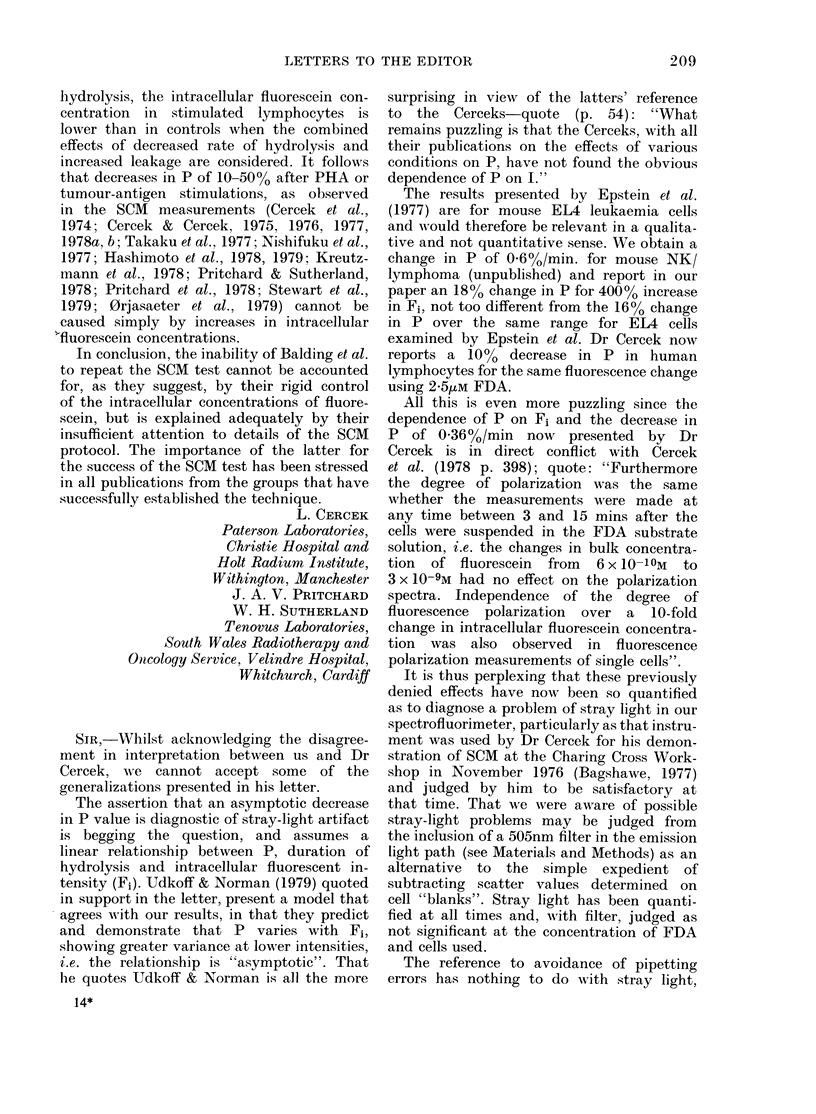# Comments on “Response of Human Lymphocytes to PHA and Tumour-Associated Antigens as Detected by Fluorescence Polarization”

**Published:** 1980-07

**Authors:** L. Cercek, J. A. V. Pritchard, W. H. Sutherland


B. CERCEK

*Paterson Laboratories,*

*Christie Hospital and*

*Holt Radium Institute,*

*Manchester*

SIR,—We are perplexed that Balding *et al.*, in their recent paper (*Br. J. Cancer*, (1980) **41,** 73) overlooked the fact that the successful accomplishment of the SCM test depends on the isolation of a specific subpopulation of lymphocytes (Cercek & Cercek, 1977, 1978*a*, *b*; Takaku *et al.*, 1977; Hashimoto *et al.*, 1978; Kreutzmann *et al.*, 1978; Pritchard and Sutherland, 1978; Pritchard *et al.*, 1978; Ørjasaeter *et al.*, 1979). In contrast to 90% pure density-specific lymphocytes (of which more than 75% are T cells) used in the SCM test, their cell preparations contained an average of only 67% lymphocytes with an undefined proportion of T cells. It is obvious that the experimental conditions for the isolation of SCM responders were not strictly followed. For example, their "cell preparations b" were isolated at 13°C instead of at 16·7°C (calculated from their data on the osmolality and density of the Ficoll–Triosil solution) resulting in the isolation of SCM-non-responding lymphocytes (Cercek & Cercek, 1978*b*). Other critical deviations from the SCM protocol include the use of HEPES instead of phosphate buffer in their "cell preparations a" as well as the use of acetone in the preparation of the FDA substrates. Both HEPES buffer (unpublished results) and impurities in the acetone diminish or abrogate SCM responses to PHA and cancer antigens (Cercek & Cercek, 1976, 1977). Furthermore, they claim that on PHA stimulation of lymphocytes the rate of fluorescein diacetate (FDA) hydrolysis increses and the leakage of fluorescein decreases. In contrast, a detailed study on the effects of PHA on the kinetics of FDA hydrolysis in human lymphocytes (Mullen & Campbell, 1979) and the data of Cercek & Cercek (1978*a*) show that the rate of FDA hydrolysis decreases on PHA stimulation by an average of 40%. It has also been reported that binding of mitogens such as PHA and Con-A to the plasma membrane of lymphocytes increases its fluidity and permeability (Greaves & Janossy, 1972; Collard *et al.*, 1977). To this we can add our own observations from hundreds of measurements on SCM-responding lymphocytes from healthy donors, cancer patients or donors with other diseases, which show that after PHA stimulation the permeation of fluorescein always increases by 5–150% according to the batch of PHA, over that of the controls. Therefore, at a constant time of FDA

hydrolysis, the intracellular fluorescein concentration in stimulated lymphocytes is lower than in controls when the combined effects of decreased rate of hydrolysis and increased leakage are considered. It follows that decreases in P of 10–50% after PHA or tumour-antigen stimulations, as observed in the SCM measurements (Cercek *et al.*, 1974; Cercek & Cercek, 1975, 1976, 1977, 1978*a*, *b*; Takaku *et al.*, 1977; Nishifuku *et al.*, 1977; Hashimoto *et al.*, 1978, 1979; Kreutzmann *et al.*, 1978; Pritchard & Sutherland, 1978; Pritchard *et al.*, 1978; Stewart *et al.*, 1979; Ørjasaeter *et al.*, 1979) cannot be caused simply by increases in intracellular fluorescein concentrations.

In conclusion, the inability of Balding *et al.* to repeat the SCM test cannot be accounted for, as they suggest, by their rigid control of the intracellular concentrations of fluorescein, but is explained adequately by their insufficient attention to details of the SCM protocol. The importance of the latter for the success of the SCM test has been stressed in all publications from the groups that have successfully established the technique.

L. Cercek
*Paterson Laboratories,*
*Christie Hospital and*
*Holt Radium Institute,*
*Withington, Manchester*
J. A. V. Pritchard
W. H. Sutherland
*Tenovus Laboratories,*
*South Wales Radiotherapy and*
*Oncology Service, Velindre Hospital,*
*Whitchurch, Cardiff*